# Menadione as Antibiotic Adjuvant Against *P. aeruginosa*: Mechanism of Action, Efficacy and Safety

**DOI:** 10.3390/antibiotics14020163

**Published:** 2025-02-07

**Authors:** Kristela Shehu, Marc Schneider, Annette Kraegeloh

**Affiliations:** 1 Department of Pharmacy, Biopharmaceutics & Pharmaceutical Technology, Saarland University, 66123 Saarbrücken, Germany; kristela.shehu@leibniz-inm.de; 2 INM—Leibniz Institute for New Materials, 66123 Saarbrücken, Germany

**Keywords:** menadione, azithromycin, *Pseudomonas aeruginosa*, mechanism of action, lung toxicity

## Abstract

**Background/Objectives:** Antibiotic resistance in chronic lung infections caused by *Pseudomonas aeruginosa* requires alternative approaches to improve antibiotic efficacy. One promising approach is the use of adjuvant compounds that complement antibiotic therapy. This study explores the potential of menadione as an adjuvant to azithromycin against planktonic cells and biofilms of *P. aeruginosa*, focusing on its mechanisms of action and cytotoxicity in pulmonary cell models. **Methods:** The effect of menadione in improving the antibacterial and antibiofilm potency of azithromycin was tested against *P. aeruginosa*. Mechanistic studies in *P. aeruginosa* and *AZMr-E. coli DH5α* were performed to probe reactive oxygen species (ROS) production and bacterial membrane disruption. Cytotoxicity of antibacterial concentrations of menadione was assessed by measuring ROS levels and membrane integrity in Calu-3 and A549 lung epithelial cells. **Results:** Adding 0.5 µg/mL menadione to azithromycin reduced the minimum inhibitory concentration (MIC) by four-fold and the minimum biofilm eradication concentration (MBEC) by two-fold against *P. aeruginosa*. Adjuvant mechanisms of menadione involved ROS production and disruption of bacterial membranes. Cytotoxicity tests revealed that antibacterial concentrations of menadione (≤64 µg/mL) did not affect ROS levels or membrane integrity in lung cell lines. **Conclusions:** Menadione enhanced the efficacy of azithromycin against *P. aeruginosa* while exhibiting a favorable safety profile in lung epithelial cells at antibacterial concentrations. These findings suggest that menadione is a promising antibiotic adjuvant. However, as relevant data on the toxicity of menadione is sparse, further toxicity studies are required to ensure its safe use in complementing antibiotic therapy.

## 1. Introduction

The relentless rise of antibiotic resistance has become a health and economic burden and is estimated to cause 10 million deaths annually by 2050 if no proper action is taken [1,2]. In the realm of respiratory health, the emergence of resistant pathogens challenges the treatment of lung diseases, which are often chronic and require frequent antibiotic therapy. Particularly, cystic fibrosis (CF), a rare genetic lung disease, has a high propensity for persistent and chronic bacterial infections, making patients susceptible to antibiotic resistance [3,4]. CF lungs, characterized by a dense and thick mucus layer, provide a fostering microenvironment for infections with pathogens such as *Pseudomonas aeruginosa*, *Staphylococcus aureus*, and *Burkholderia cenocepacia* [5]. *P. aeruginosa* remains the most prevalent opportunistic Gram-negative pathogen that colonizes the CF lung, and it is classified by the World Health Organization as a critical priority microorganism for which there is a pressing need for treatment [6,7]. The treatment of *P. aeruginosa* infections in CF includes a combination of inhaled, oral, and i.v. antibiotics like tobramycin, aztreonam, and colistimethate sodium [3,8]. In addition, azithromycin (AZM) has been reported to impair *P. aeruginosa* growth, mainly due to its anti-inflammatory, anti-virulence, and anti-biofilm properties [9,10].

Over the years, these antibiotics have been pivotal in managing *P. aeruginosa* infections in CF patients. Although they have indeed improved the disease prognosis, due to the chronic and recurrent nature of these infections, as well as the long treatment, they can favor the development of bacterial resistance, making the treatment even more difficult as the disease progresses [11,12]. Therefore, the evolution of bacterial resistance demands a shift towards new approaches for tackling these infections and breaking the resilience cycle. There are several methodologies being exploited to cope with antibiotic resistance, such as semi-synthetic engineering, quorum-sensing inhibitors, phage therapy, or antimicrobial peptides and nanoparticles [13]. One promising approach involves using adjuvants that complement antibiotic therapy by enhancing the efficacy of antibiotics through synergistic mechanisms.

In this context, 1,4-Naphthoquinones (NQ), a group of organic compounds which exhibit diverse biological activities that range from antimicrobial, antifungal, antiviral, and antitumoral have gained attention for their potential in complementing antibiotic therapy [14,15]. They can either be found in natural resources or synthesized and have been studied for their potential as adjuvants to antibiotics through mechanisms such as the inhibition of efflux pumps, generation of reactive oxygen species (ROS), inhibition of DNA gyrases, inhibition of biofilms, and disturbance of bacterial membrane [16,17,18].

Menadione, also known as vitamin K3, is a synthetic 1,4-NQ derivative, substituted at position 2 by a methyl group. Its antibacterial activity has been demonstrated against Gram-negative pathogens such as *P. aeruginosa*, *E. coli*, and *K. pneumoniae*, as well as against Gram-positive bacteria like *S. aureus* and *Streptococcus pneumoniae* [19,20]. Additionally, it has been exploited for its potential to increase the activity of antibiotics ampicillin, ciprofloxacin, and oxacillin against methicillin-resistant and sensitive *S. aureus* strains [21,22]. Menadione can induce the production of ROS through redox cycling, resulting in the damage of proteins, lipids, DNA, and other cellular components [23]. It has been proposed that the induction of ROS is one of the main mechanisms associated with the antibacterial activity of menadione [21]. On the other hand, given its highly lipophilic nature, menadione is accustomed to incorporating into cell membranes and enhancing membrane fluidity, thereby modulating its permeability and the activity of membrane proteins [24]. The tendency of menadione to interact with cell membranes and damage their integrity has been suggested as a potential adjuvant mechanism when combined with antibiotics [19,24]. However, these mechanistic aspects of menadione have been studied mainly in Gram-positive bacteria, and there are few reports in the literature regarding its mechanism of action in Gram-negative pathogens such as *P. aeruginosa*.

However, even though menadione has garnered considerable interest due to its potential antibacterial properties [19], the cytotoxicity of antibacterial concentrations of menadione, in relevant eukaryotic cell models, has been poorly investigated. As there has been evidence about menadione-induced hepatotoxicity and other damage related to oxidative stress, its cytotoxicity should be taken into consideration and further evaluated when it comes to using this compound as an antibiotic adjuvant [25].

In this study, we investigate the ability of menadione to enhance the activity of azithromycin against planktonic cells and biofilms of *P. aeruginosa* and elucidate mechanistic aspects that lie behind its adjuvant properties by using an azithromycin-resistant *E. coli* DH5α as a model strain. First, the antimicrobial and antibiofilm activity of azithromycin and menadione, separately and combined together, were determined. Second, the mechanism of action of menadione in both strains was probed by measuring the generation of ROS species and the release of mCherry from cells. Finally, the impact of menadione at antibacterial concentrations, alone and in combination with azithromycin, was evaluated in two lung epithelial cell lines, Calu-3 and A549, representing the bronchial and alveolar epithelium, respectively.

## 2. Results and Discussion

### 2.1. Antibacterial Activity of Menadione and Azithromycin Against Planktonic Bacteria

Menadione has shown antibacterial activity against planktonic Gram-positive bacteria such as *S. aureus* and *S. pneumoniae* as well as against Gram-negative pathogens such as *P. aeruginosa*, *E. coli* and *K. pneumoniae* [15,19,22,26]. Its reported inhibitory concentration varies from 64 to 1024 µg/mL, depending on the bacterial strain and the growth conditions. Moreover, menadione has also been investigated as a complementary compound to known antibiotics, to potentially enhance their activity. Recent reports indicate the potential of menadione to strengthen the activity of fluoroquinolone, aminoglycoside, and ß-lactam antibiotics, mainly against multi-drug resistant (MDR) *S. aureus* [21,22]. So far, the ability of menadione to potentiate the activity of macrolide antibiotics, such as azithromycin, in Gram-negative bacteria like *P. aeruginosa*, has not been reported. Azithromycin exerts its mechanism inside the bacterial cells, where it inhibits protein synthesis by targeting the 50S subunit of the ribosome [27]. In this context, menadione is assumed to improve the intracellular availability of the drug by pore formation or change of membrane fluidity [24]. AZM is not approved yet for the treatment of *P. aeruginosa* infections. However, some clinical studies have shown that patients profit from treatment with AZM when suffering from persistent *P. aeruginosa* infections, in diseases like cystic fibrosis, chronic obstructive pulmonary disease (COPD), or diffuse panbronchiolitis (DPB) [28,29]. Other inhibitory mechanisms of AZM in *P. aeruginosa* are assumed to be the suppression of bacterial motility and inhibition of many proinflammatory, persistence-promoting virulence factors as well as targeting quorum sensing [9,29].

We determined the minimum inhibitory concentration (MIC) of menadione and azithromycin, separately and combined with each other, against azithromycin-resistant AZMr-*E. coli* DH5α and against sensitive *P. aeruginosa* strains (Table 1). Both menadione and azithromycin showed inhibitory activity when tested individually against the bacterial strains. Menadione showed an MIC of 64 µg/mL and 62.5 µg/mL in AZMr-*E. coli* DH5α and *P. aeruginosa*, respectively (Table 1). Azithromycin exhibited an MIC of 512 µg/mL and 62.5 µg/mL in AZMr-*E. coli* DH5α and *P. aeruginosa*, respectively (Table 1). However, when combined, via the checkerboard design, a four-fold reduction in the inhibitory concentration of AZM towards both strains was observed (15/0.5 µg/mL for *P. aeruginosa* and 128/16 µg/mL for AZMr-*E. coli* DH5α). Hence, we concluded that, at concentrations below its MIC, menadione behaves as an adjuvant to azithromycin, potentiating its activity and resulting in a lower inhibitory concentration of azithromycin.

**Figure 1:**
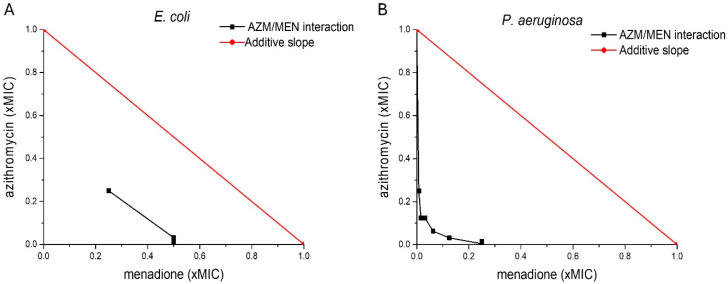
FICI isobolograms for inhibitory combinations of azithromycin and menadione (AZM/MEN) in (**A**) AZMr-*E. coli* DH5α and (**B**) *P. aeruginosa*. The red line represents the additive effect (known as the additive slope) and deviations from additivity below or above the red slope, are indicative of synergism or antagonism between two drugs, respectively. The black lines in the graphs represent the interactions between azithromycin and menadione, which are synergistic, as they lie below the additive slope for both strains investigated. The axes represent the fraction of the MIC concentration that contributes to the inhibition of the bacterial growth in the combination, for both drugs, respectively.

We further investigated the nature of the interaction between azithromycin and menadione, by calculating the fractional inhibitory concentration index (FICI) of AZM/MEN combinations. The FICI value is considered a standard reference parameter to evaluate the nature of drug interactions between two antimicrobial agents [30,31]. It provides insights into whether the combination of two drugs enhances or hinders their individual effectiveness. This way, FICI helps guide the use of antibiotic combinations in treating infections, especially in MDR pathogens or biofilm-associated infections, where single drugs might fail [31]. The FICI of AZM/MEN combinations was calculated based on Loewe’s additivity model [31]. The additive effect is plotted as a red line (additive slope) in Figure 1. Any line below this red line hints towards a synergistic interaction between two compounds [30]. FICI isobolograms [32] indicated that in both strains, the interaction between menadione and azithromycin was of a synergistic nature, with calculated FICI values of 0.248 for *P. aeruginosa* and 0.5 for AZMr-*E. coli* DH5α, as reported in Table 1. In the literature, drug interactions are defined as synergistic if FICI ≤ 0.5, no interaction if 0.5 < FICI ≤ 4, and antagonistic if FICI > 4 [31].

To assess if there is any interaction between azithromycin and menadione after mixing at antibacterial concentrations, an LC/MS analysis was performed (Supplementary Information, Figure S1). As the retention times (RTs) of azithromycin and menadione in the mixture corresponded precisely to their respective standards and no additional peaks were observed in the mixture, we concluded that no chemical reactions, e.g., the formation of adducts, occurred between azithromycin and menadione under the tested conditions.

### 2.2. Activity of Menadione and Azithromycin Against P. aeruginosa Biofilms

Besides targeting planktonic bacteria, biofilm formation poses a major therapeutic challenge due to the reduced metabolic activity of the bacteria, impaired antibiotic penetration and the enzymatic deactivation of drugs within the biofilm matrix [33]. Given these factors, it is crucial to evaluate the antibiofilm potency of a drug, as biofilm-associated infections are more resistant to conventional treatments.

#### 2.2.1. Minimum Biofilm Eradication Concentration (MBEC)

Biofilms are matrix-enclosed biotic and abiotic microbial aggregates that form structurally complex and dynamic systems and allow cells to survive in hostile environments [34]. *P. aeruginosa* is well known for forming resilient biofilms that survive in several environments, including the cystic fibrosis lung [7].

The ability of menadione and azithromycin to affect mature biofilms of *P. aeruginosa* was evaluated by using the Calgary Biofilm device (CB), commercially available as the MBEC assay system [35]. This system is made of two components: the top component is a lid that has 96 pegs attached to it, which are designed to fit into the wells of a standard 96-well plate (bottom component) [35]. Using this system, bacterial biofilms are grown on the surface of peg lids, wherein the presence of shear forces, bacteria become attached to the surface of the pegs and form with time mature biofilms [36]. We allowed biofilms of *P. aeruginosa* to mature on the surface of the pegs for two and a half days and then exposed them to azithromycin and menadione, separately and combined with each other, to determine the minimum biofilm eradication concentration (MBEC). Table 2 depicts the MBEC of azithromycin (256 µg/mL) and menadione (512 µg/mL). In contrast to planktonic *P. aeruginosa* cells, menadione showed limited activity against mature biofilms of *P. aeruginosa*. This poor antibiofilm potency of menadione against *P. aeruginosa* is not yet described in the literature. Current studies mainly describe its antibiofilm action against Gram-positive bacteria such as *S. aureus.* For instance, Mone et al. reported that MEN successfully inhibited the biofilm formation as well as disrupted the pre-grown biofilms of different strains of *S. aureus*, at its respective MIC values [37]. Leitao et al. have also reported the antibiofilm potency of MEN against growing and pre-formed biofilms of *S. aureus*, alone or in combination with oxacillin [22]. Both Gram-positive and Gram-negative bacteria can form biofilms [38]. Although biofilms formed by bacteria of these two groups share physical, chemical, and regulatory characteristics [38], specific processes involved in biofilm formation may differ between these two groups. For example, quorum sensing, a process of cell–cell signaling in biofilms, differs in these two types of bacteria [39]. This could explain why menadione has shown a strong antibiofilm potency against *S. aureus* biofilms [22,37] but exhibited poor activity in our experiments against biofilms of *P. aeruginosa*. In addition, biofilm formation is a complex process with several steps and factors that might impact the outcome, like the bacterial strain and growing conditions, which should be taken into consideration when interpreting the antibiofilm activity.

**Figure 2:**
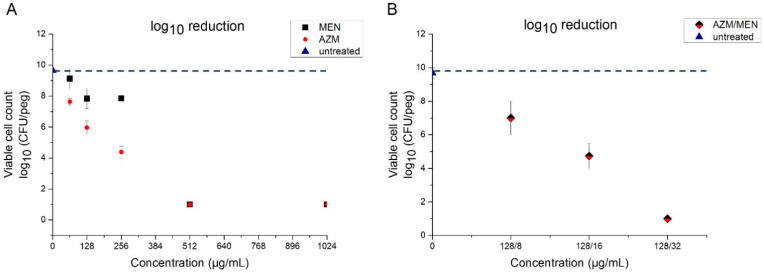
Viable count expressed as colony forming units (CFU) in (**A**) biofilms treated with different concentrations (128–1024 µg/mL) of azithromycin or menadione and (**B**) biofilms treated with different combinations of azithromycin and menadione. In both cases, the values are compared with untreated biofilms (pegs). Values below the dashed line indicate the reduction in the bacterial population compared to the untreated pegs. Graph A shows the stronger impact of AZM on the viability of *P. aeruginosa* in the biofilm compared to menadione when applied at the same mass concentration. When combined (**B**), menadione strengthens the effect of azithromycin on viability, which again highlights the adjuvant effect of menadione also for the treatment of *P. aeruginosa* biofilms.

In contrast to menadione, azithromycin showed stronger activity against *P. aeruginosa* biofilms, as indicated by the lower MBEC value (Table 2). The determined MBEC was in accordance with the values reported by San Mauro et al., who also used the CB device to test the antibiofilm potency of AZM [40]. Moreover, viable counts revealed that AZM caused a higher log_10_ reduction as compared to menadione (Figure 2A). Finally, we could observe an adjuvant effect of menadione to azithromycin, also against biofilms of *P. aeruginosa*. When combined with menadione (azithromycin/menadione), azithromycin exhibited a lower MBEC, whereby, after adding 16 µg/mL menadione, a two-fold reduction in the antibiofilm concentration of azithromycin alone was achieved (Table 2). The log_10_ reduction achieved at the MBEC combination (128/16 µg/mL) (Figure 2B) was similar to the log_10_ reduction achieved at MBEC of azithromycin alone (256 µg/mL). This indicates that, even though menadione did not exhibit strong antibiofilm properties on its own, it showed an adjuvant effect to azithromycin in eradicating biofilms of *P. aeruginosa*.

The activity of AZM and MEN in killing the dispersed planktonic cells that have been shed from the biofilms was also determined. The minimum bactericidal concentrations (MBCs) were 128 µg/mL for azithromycin, 256 µg/mL for menadione, and 128/8 µg/mL for their combination (Supplementary Information, Figure S2).

#### 2.2.2. Morphology of P. aeruginosa Biofilms

##### Untreated *P. aeruginosa* Biofilms

**Figure 3:**
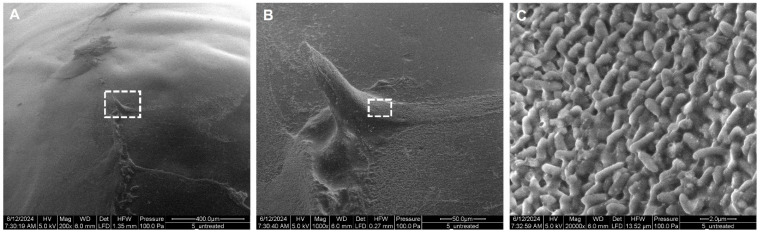
Scanning electron microscopy images of a mature *P. aeruginosa* biofilm grown for two and a half days at the surface of the pegs (untreated): (**A**) Overview of the biofilm surface (scale bar 400 µm). (**B**) Magnification (×5) of the area indicated by the dashed box in image (**A**) (scale bar 50 µm), showing smooth and rough areas of the biofilm. (**C**) Magnification (×20) of the area indicated by the dashed box in image (**B**), showing single cells present on top of the biofilm (scale bar 2 µm).

After two and a half days in the incubator, a relatively thick and viscous, light-green biofilm of *P. aeruginosa*, attached to the tip of the pegs, could be observed by the bare eye. Biofilms covered, as expected, about 30–40% of the whole peg [36]. Figure 3 reveals the morphology of untreated biofilms captured via scanning electron microscopy (SEM). In Figure 3A, we can observe a gel-like matrix representing the biofilm on the surface of the peg. At a higher magnification, the three-dimensional structure of the biofilm, with rough and smooth structures, could be observed (Figure 3B). Further magnification (Figure 3C) revealed that the observed structures are composed of densely packed bacteria (*P. aeruginosa* rods, about 1–2 µm in length) attached to each other via strings of extracellular polymeric substance (EPS).

##### Azithromycin- and Menadione-Treated *P. aeruginosa* Biofilms

**Figure 4:**
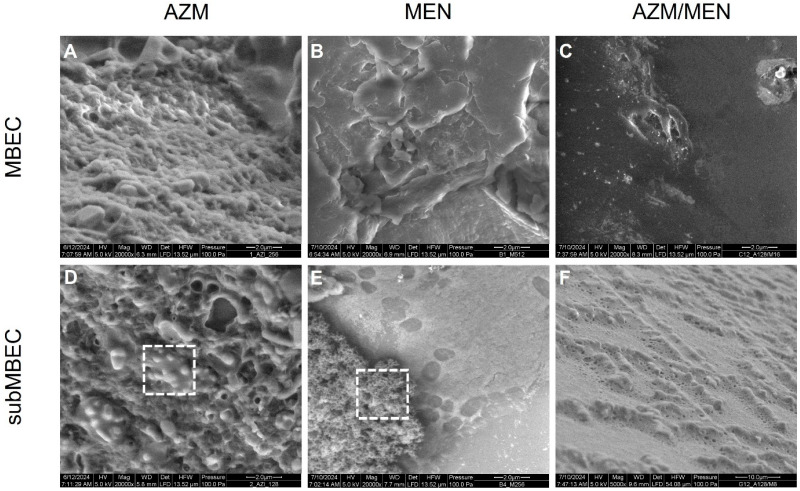
Scanning electron microscopy images of *P. aeruginosa* biofilms treated with MBEC concentrations of azithromycin (256 µg/mL), menadione (512 µg/mL), and azithromycin/menadione in combination (128/16 µg/mL) (**A**–**C**) and their subMBEC concentrations (**D**–**F**). Images show that treatment at the MBEC concentrations completely eradicates the bacteria, leaving only debris of the biofilms at the surface of the pegs. Meanwhile, when treated with subMBEC concentrations, bacteria can still be observed attached to the biofilm, as indicated by the dashed boxes in (**D**,**E**). The treatment with the combination of AZM/MEN eradicates bacteria at both MBEC and subMBEC concentrations, underlying the stronger impact that the addition of menadione to azithromycin has.

The impact of azithromycin and menadione, separately and combined, on the morphology of mature *P. aeruginosa* biofilms can be observed in Figure 4. Treatment with AZM and MEN at their MBEC concentrations seems to leave only remnants of EPS and salts at the surface of the pegs, with no visible *P. aeruginosa* cells embedded in the EPS, further supporting the eradication of bacteria at the determined MBEC concentrations (Figure 4A,B). Moreover, the appearance of these EPS debris seems to be different in the biofilms treated with azithromycin as opposed to those treated with menadione. In Figure 4A, the EPS seems more undulating compared to the flatter and compact EPS shown in Figure 4B. On the other hand, when treating the biofilms with subMBEC concentrations of azithromycin and menadione (Figure 4D,E), bacterial cells were not fully eradicated but still enclosed within the extracellular matrix. The bacterial density appeared, however, to be lower in the biofilms treated with azithromycin (D) in contrast to those treated with menadione, where denser and aggregated clumps of *P. aeruginosa* cells could be observed, as indicated by the dashed box in (E).

Combined, azithromycin, and menadione (128/16 µg/mL) seem to fully eradicate the bacterial cells in the biofilm and leave less debris of the EPS, as opposed to when given separately (Figure 4C). The presence of bacterial cells in the biofilms was less or not observed at all, even at the subMBEC concentration of AZM/MEN (F), which highlights again the stronger impact that the combination of azithromycin and menadione has compared to each of them individually.

### 2.3. Antibacterial Mechanisms of Menadione

#### 2.3.1. Induction of Reactive Oxygen Species

**Figure 5:**
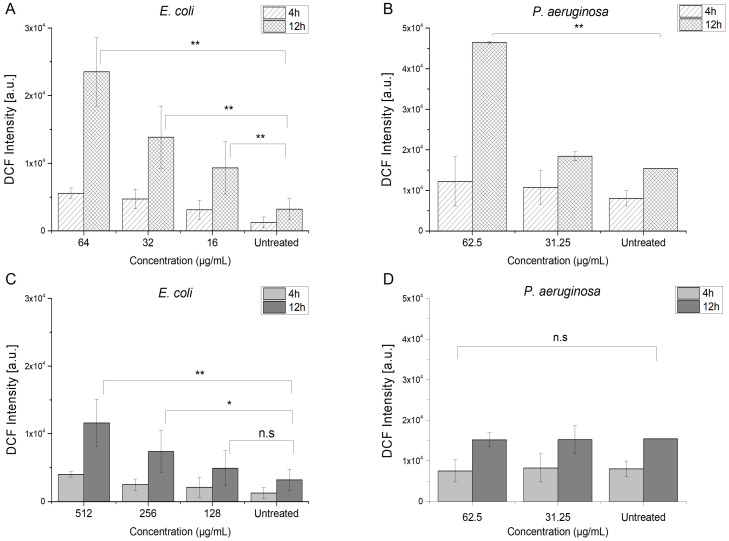
ROS generation induced by MIC and subMIC concentrations of menadione (**A**,**B**) and azithromycin (**C**,**D**) after treatment of AZMr-*E. coli* DH5α and *P. aeruginosa* for 4 and 12 h. Treatment with menadione shows a concentration- and time-dependent production of ROS in both strains. Data represent mean values ± SE for *n* = 9 from 3 independent experiments. The significance of the obtained data is calculated with One Way ANOVA followed by the Bonferroni test and indicated by asterisks: *, *p* < 0.05 (*p* values ranging from 0.01496–0.04408); **, *p* < 0.01 (*p* values ranging from 0.00225–0.00921); n.s, non-significant.

Intracellular oxidants, when at low concentration, play a role as redox-active messengers in signal transduction pathways that respond to various stimuli such as growth factors, hypoxia, etc. However, durable elevated levels of oxidative stress can cause oxidative damage to lipids, proteins, RNA, and DNA, ultimately resulting in cell death through apoptosis and/or necrosis [41]. Menadione is a polycyclic aromatic ketone that has the potential to undergo a process of one-electron reduction, leading to the formation of a semiquinone radical. Subsequently, this semiquinone radical can reduce molecular oxygen, resulting in the production of superoxide anion radicals, while itself returning to the initial quinone form. This futile cycle of redox reactions generates intracellular reactive oxygen species (ROS), which can rapidly oxidize biological molecules in both the mitochondrial matrix and cytosol [24]. We determined the generation of ROS species in AZMr-*E. coli* DH5a and *P. aeruginosa* upon treatment with MIC and subMIC concentrations of menadione and azithromycin (Figure 5).

*Menadione*. Figure 5A,B shows that MEN induced the generation of ROS species in both AZMr-*E. coli* DH5α and *P. aeruginosa* at its respective MIC and subMIC concentrations. We could observe concentration-dependent ROS production in both strains. Moreover, the generation of ROS increased significantly by increasing the treatment time to 12 h, as indicated by the signal of the fluorescent probe, DCF. The ROS-inducing effect of menadione seems to be stronger in AZMr-*E. coli* DH5α, where the treatment with subMIC concentrations also caused a significant ROS production compared to the untreated cells.

The induction of ROS by menadione has already been linked to its antibacterial activity in literature. Mone et al. explored the mechanism of action of MEN against MDR *S. aureus*. Their findings indicated that MEN increased oxidative stress in several *S. aureus* strains at an MIC of 64–256 μg/mL after 6 h, with the effects persisting for up to 12 h [21]. Another study shows that menadione induced moderate ROS production at its MIC against *S. aureus*; however, when combined with oxacillin, ROS production is doubled [22]. Schlievert et al. also reported the induction of ROS, including superoxide anions, in all the tested strains of *S. aureus*. Interestingly, they show that menadione is also bactericidal against *S. aureus* grown under anaerobic conditions, though this was a weaker effect compared to its activity in an aerobic environment [42]. In general, our results align with the reported ROS-inducing behavior of menadione, in relation to its antibacterial properties.

*Azithromycin*. Azithromycin, on the other hand, is not primarily known for generating ROS as its mechanism of action. Instead, AZM primarily works by inhibiting bacterial protein synthesis by binding to the 50S ribosomal subunit, thereby interfering with the translocation steps in protein synthesis [9]. However, we observed ROS generation in AZMr-*E. coli* DH5α upon treatment with MIC and subMIC concentrations of azithromycin (Figure 5C). Treatment with AZM showed a concentration- and time-dependent significant ROS generation in this strain. On the other hand, this effect was not observed in *P. aeruginosa* (Figure 5D). The involvement of ROS in antibiotic-mediated killing remains unresolved in the scientific community. There are technical and biological arguments against the involvement of ROS, along with numerous studies which have indeed reported evidence of ROS induction upon antibiotic treatment, using chemiluminescence or fluorescence-based techniques [43]. Hong et al. demonstrated the killing of *E. coli* cells due to the stimulation of self-amplifying ROS, which exceeded the capacity of bacteria to repair the primary damage caused by the tested antibiotics [44]. If the primary damage of the antibiotic is not strong enough to kill the bacteria (damage related to the interaction of the antibiotic with the cellular target), this could lead to the induction of ROS species by activating the tricarboxylic acid cycle [43]. This ROS-mediated damage may trigger further ROS accumulation, creating a self-amplifying and unstoppable process, which prompts the final cell death [44]. This could explain why we observed ROS generation in AZMr-*E. coli* DH5α and not in *P. aeruginosa*. *P. aeruginosa* was more sensitive to azithromycin, compared to the AZMr-*E. coli* DH5α, as indicated by the difference in the MIC values (62.5 and 512 µg/mL, respectively). As the strain is resistant to azithromycin, the primary damage of AZM might not be strong enough to immediately lead to cell death, causing this generation of ROS, which further contributes to the action of azithromycin.

**Figure 6:**
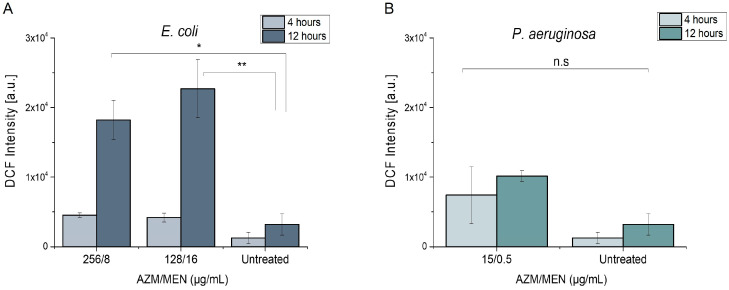
ROS generation induced by combined inhibitory concentrations of azithromycin and menadione (AZM/MEN) after treatment for 4 and 12 h of (**A**) AZMr- *E. coli* DH5α and (**B**) *P. aeruginosa.* Data represent mean values ± SE for *n* = 9 from 3 independent experiments. The significance of the obtained data is calculated with One Way ANOVA followed by the Bonferroni test and indicated by asterisks: *, *p* < 0.05 (*p* = 0.01064); **, *p* < 0.01 (*p* = 0.00276); n.s, non-significant.

*Azithromycin and menadione.* Figure 6 reveals the generation of ROS species in AZMr- *E. coli* DH5α and *P. aeruginosa* upon treatment with a combination of AZM and MEN at their MIC concentrations. We could see that, in AZMr-*E. coli* DH5α, the ROS generation increased after increasing the treatment time to 12 h, and it is mainly determined by the concentration of MEN in the combination (Figure 6A). Meanwhile, in *P. aeruginosa*, ROS formation could be observed when cells were treated with AZM/MEN compared to the untreated sample, albeit the overall ROS production was lower and non-significant (Figure 6B). This might be explained by the low concentration of MEN in the combination (0.5 µg/mL).

The generation of ROS plays a significant role in the mechanism by which menadione enhances the antibiotic effect of azithromycin, as observed in the results described. In *AZMr-E. coli* DH5α, the ROS production increased over time, driven predominantly by the concentration of MEN in the combination. This indicates that ROS formation contributes significantly to the antibacterial effect of the combination, as oxidative stress compromises bacterial cellular components, including membranes and proteins, enhancing the susceptibility of *E. coli* to azithromycin’s action. In *P. aeruginosa*, while ROS formation was less pronounced and non-significant due to the low concentration of MEN (0.5 µg/mL), the observed ROS levels still support the potential for ROS to contribute to membrane destabilization and increased permeability, thereby facilitating azithromycin uptake. ROS produced by menadione weakens the bacterial membrane by causing lipid peroxidation, leading to increased permeability and possible improved entry of azithromycin into the bacterial cell, enhancing its intracellular concentration and activity. Moreover, ROS caused by MEN might impair bacterial defense systems, such as efflux pumps or biofilm barriers, which are common resistance mechanisms in *P. aeruginosa*. By disabling these defenses, azithromycin becomes more effective at lower doses.

#### 2.3.2. Disturbance of Bacterial Membrane Integrity

The role of menadione in inducing the formation of ROS in Gram-positive bacteria is already described in the literature. Our data indicate that ROS generation also contributed to the antibacterial activity of MEN against both Gram-negative strains tested in this study. However, it has been mentioned that ROS generation might not be the only mechanism involved in the antibacterial properties of menadione. Given its lipophilic nature, MEN has the potential to incorporate into cell membranes, thus promoting alterations of membrane physical properties, such as membrane fluidity [24]. Monteiro et al. demonstrated MEN incorporation into membranes by using liposomes of different compositions and isolated mitochondrial membranes and measuring the released Calcein levels, indicating altered membrane permeability [24]. Further studies indicate that disturbance of the bacterial membrane might finally cause cell death and that Gram-negative bacteria are more affected than Gram-positive ones [19]. Nevertheless, this possible antibacterial mechanism remains, so far, an assumption.

Therefore, we assumed that, in case MEN damages the bacterial membrane, it may create pathways or pores, through which intracellular components can leak from the damaged cell into the supernatant. To investigate this, we used the AZMr-*E. coli* DH5α, intracellularly expressing the mCherry protein, as a model Gram-negative strain to probe possible bacterial membrane damage caused by MEN [45]. mCherry is a monomeric red fluorescent protein with a theoretical molecular weight of 28 kDa, derived from the *Discosoma* sp. fluorescent protein “DsRed” [46]. It has been used as an accessory protein to demonstrate cell membrane injury, for instance, in myoblasts or the release of damage-associated molecular pattern (DAMP) molecules upon membrane rupture [47,48].

**Figure 7:**
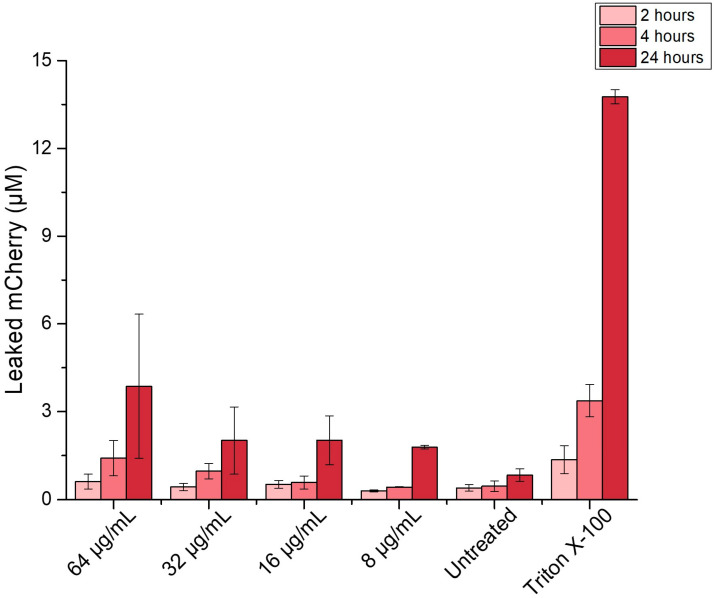
MIC (64 µg/mL) and subMIC concentrations of menadione cause leakage of mCherry protein from the cells indicating disturbance of the membrane of AZMr- *E. coli* DH5α. Samples lysed with Triton X-100 were included as a positive control. Data shown are mean values ± SE for *n* = 9 from 3 independent experiments.

Indeed, we could measure the fluorescence of mCherry in the supernatant, inferring the damage of bacterial membrane by the MIC and subMIC concentrations of MEN and leakage of mCherry from the cytosol, possibly via passive diffusion through opened pathways in the membrane (Figure 7). Figure 7 shows that mCherry leakage depends on the concentration of MEN (8–64 µg/mL). The concentration of mCherry in the supernatant increased with the treatment time, possibly due to the constitutive formation of mCherry. Cells lysed with 9% Triton X-100 were included as a measure for the maximum mCherry release possible. The release of mCherry into the surroundings was confirmed by the use of His-Ni affinity beads, to which mCherry binds through its C-terminal histidine tag (Supplementary Figure S3) [49]. Interestingly, beads stained positive with mCherry even when menadione was combined with an antioxidant compound such as vitamin C. Vitamin C serves as a scavenger and counteracts the MEN-induced ROS formation. However, the release of mCherry, indicated by the red beads (MEN64/VitC50), even in the presence of a scavenging compound, may imply that membrane damage occurs independently (although to a much lower extent) from ROS formation. The intensity of extracellular mCherry detected in the presence of VitC, however, appeared lower compared to the samples treated with MEN only (Supplementary Figure S3).

### 2.4. Effect of Menadione on Epithelial Lung Cells

#### 2.4.1. Impact of Menadione on Membrane Integrity of Calu-3 and A549 Cells

Menadione is gaining increasing attention due to its potential antitumor and antibacterial properties [22,37,50]. In this context, a careful evaluation of its cytotoxic profile is critical. Menadione is a pro-drug that converts to vitamin K2 in the liver, playing a role in blood coagulation and tissue calcification [51]. Therefore, it was previously approved by the FDA for oral use [52]. However, high oral (5–10 mg) and intravenous (2 mg) doses used to improve neonatal hemostasis, were linked to kernicterus, leading to its withdrawal from the market [51]. There has also been evidence of menadione-induced hepatotoxicity and additional damage associated with oxidative stress [25]. As menadione has regained scientific interest for its antimicrobial properties, the cytotoxicity of its antibacterial concentrations in relevant biological models needs to be evaluated, even though these concentrations are generally lower compared to the oral or i.v. doses used for neonatal hemostasis (µg/mL range compared to 5–10 mg oral tablets). Given that adjuvant concentrations of menadione enhanced the efficacy of azithromycin against *P. aeruginosa*, the predominant pathogen in CF lungs, we assessed as a basic step whether these concentrations exhibit cytotoxicity toward lung cells. Ideally, menadione should effectively aid azithromycin in eradicating *P. aeruginosa* without inducing cytotoxic effects on lung cells when potentially administered via inhalation. To test this, we used two pulmonary cell lines, Calu-3 and A549, which represent the bronchial and alveolar epithelium, accordingly. Calu-3 cells are a suitable in vitro model for the investigation of delivery systems intended for pulmonary use and assessment of long or repeated exposures to potentially toxic compounds, as they can produce a relatively thick mucus layer when grown at AIC conditions and can form tight junctions [53,54]. In addition, A549 cells, a model for alveolar type II cells, are frequently used for in vitro toxicity studies [55].

**Figure 8:**
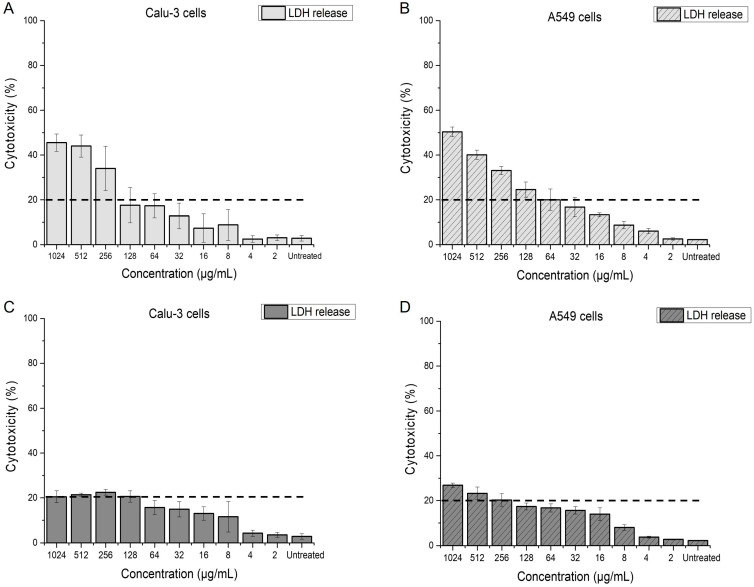
Impact of menadione (**A**,**B**) and azithromycin (**C**,**D**) on the membrane integrity of Calu-3 and A549 cells at a concentration range from 2 to 1024 µg/mL. Values below the horizontal dashed line (≤20%) are assumed to be non-cytotoxic concentrations. Graphs show that antibacterial concentrations of MEN and AZM do not induce cytotoxicity in Calu-3 and A549 cells. Data shown are mean values ± SE for *n* = 9 from 3 independent experiments.

To assess the impact of menadione and azithromycin on the membrane integrity of pulmonary cells, the lactate dehydrogenase (LDH) based membrane integrity assay was performed (Figure 8). Using this assay, cytotoxicity values of ≤20%, correlating with a low release of LDH, are considered nontoxic [56]. Figure 8A,B indicates that antibacterial concentrations of menadione (≤64 µg/mL) did not affect the membrane integrity, and cytotoxicity in both cell lines was induced only at much higher concentrations (≥256 µg/mL). Azithromycin, on the other hand, did not impair the membrane integrity of the cells even when applied at concentrations two to four-fold higher than its antibacterial concentration against *P. aeruginosa* (Figure 8C,D). Overall, A549 cells seem more sensitive to azithromycin than Calu-3 cells (Figure 8D).

#### 2.4.2. ROS Induction in Calu-3 Cells

We previously demonstrated that ROS production is one of the mechanisms involved in inhibiting the growth of AZMr-*E. coli* DH5α and *P. aeruginosa* cells. In addition, studies have shown that most of the reported cytotoxic effects of MEN in eukaryotic cells are predominantly attributed to oxidative damage resulting from ROS generation. MEN-induced effects further result in macromolecular damage, the disruption of calcium homeostasis, depletion of cellular thiols, and elevated lipid peroxidation [23]. McCormick et al. reported a dose-dependent increase in both intracellular calcium and ROS formation when A549 cells are exposed to menadione [57]. Some studies suggest that MEN induces programmed cell death by causing mitochondrial depolarization and the release of cytochrome c into the cytosol, as observed in pancreatic acinar cells [58]. Moreover, menadione activates apoptosis also by increasing ROS production through a redox-cycling mechanism [50]. Another study performed with cardiomyocytes suggests that menadione activates a plethora of cell death pathways, in all of which, poly (ADP-ribose) polymerase-1 (PARP-1), a nuclear enzyme that plays a critical role in DNA repair, genomic stability, and cellular response to stress, is involved [41].

**Figure 9:**
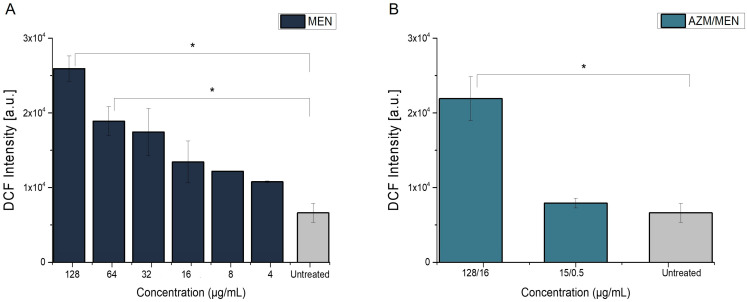
Induction of ROS in Calu-3 epithelial cells by antibacterial concentrations of menadione alone (**A**) and menadione combined with azithromycin (**B**). Significant ROS production is observed in cells treated at MIC and higher concentrations of menadione after a 24-h treatment (**A**). ROS formation appears to be dependent on the concentration of menadione and non-significant for subMIC and lower concentrations as compared to the negative control. The combination of azithromycin and menadione (**B**), which was found inhibitory in AZMr-*E. coli* DH5α (128/16 µg/mL), shows significant ROS generation in Calu-3 cells. In contrast, when treated with the combination of azithromycin and menadione (15/0.5 µg/mL) (inhibitory against *P. aeruginosa*), the generation of ROS is non-significant, as indicated by the low DCF intensity. Data shown are mean values ± SE for *n* = 6 from 3 independent experiments. Significance of the obtained data is calculated with One Way ANOVA followed by the Bonferroni test and indicated by asterisks: *, *p* < 0.05 (*p* values ranging from 0.01162–0.04051).

We tested whether the determined antibacterial concentrations of MEN induce any ROS-related damage in Calu-3 cells, which are derived from a bronchial adenocarcinoma. Calu-3 cells were grown at AIC conditions for 21 days in order to allow them to form a tight epithelium and were treated with different concentrations of MEN for 24 h from the basolateral side of the transwell in order to circumvent the protective function of the mucus layer and expose the cells directly to MEN [54,59]. Figure 9A reveals a significant ROS generation in Calu-3 cells when they were exposed to ≥ MIC concentrations of MEN (≥ 64 µg/mL). Moreover, ROS formation appeared to be dependent on the concentration of MEN and non-significant for subMIC concentrations, as compared to the negative control. On the other hand, when exposing the cells to the combined treatment, at the inhibitory concentration of 15/0.5 µg/mL against *P. aeruginosa*, MEN did not significantly elevate the level of ROS in the cells, possibly due to its low concentration in the combination (only 0.5 µg/mL) (Figure 9B). In contrast, when cells were treated with 128/16 µg/mL azithromycin and menadione, the inhibitory combination against AZMr-*E. coli* DH5α and ROS generation was significantly higher (Figure 9B).

## 3. Materials and Methods

### 3.1. Bacterial Strains

In this study, two bacterial strains were used to determine the potential and mechanism of menadione as an antibacterial activity enhancer in combination with azithromycin. In the first step, a non-pathogenic, drug-resistant *E. coli* DH5α strain was used as a model strain for elucidating the adjuvant mechanism of menadione (New England BioLabs, Frankfurt, Germany) [60]. This strain was harboring plasmid *pLp3050sNuc* (*Addgene* plasmid # 122030), which contains an erythromycin resistance cassette (ermB) [61]. In addition, the strain was also engineered to heterologously express mCherry as a fluorescent reporter, under the control of a constitutive promoter P*_tipA_* [45]. The erythromycin methyltransferase B (ermB) demethylates a single adenine in 23S rRNA (adenine (2058)-N (6))-methyltransferase Erm(B)) and confers the resistance to macrolide antibiotics to the bacteria, including azithromycin [62,63,64].

*Pseudomonas aeruginosa* PAO1 (DSM 22644, German Collection of Microorganisms and Cell Cultures, Braunschweig, Germany) [65] was used to test the antimicrobial activity of azithromycin and menadione. PAO1 is the most common, non-mucoid, motile laboratory *P. aeruginosa* strain, whose genome has been fully sequenced [66]. Table 3 summarizes the information on the used strains.

### 3.2. Media and Growth Conditions

Luria Bertani (LB) medium (Carl Roth GmbH, Karlsruhe, Germany) was used to cultivate and assess the growth of AZMr-*E. coli* DH5α and *P. aeruginosa* strains [67]. LB medium was prepared at a concentration of 20 g/L, sterilized at 121 °C for 15 min and stored at room temperature. LB agar plates were prepared by adding 15 g/L agar-agar (Carl Roth GmbH, Karlsruhe, Germany) to the LB medium.

M9 minimal medium was prepared according to the recipe of Cold Spring Harbor protocols [68]. Solutions of glucose 20%, 1 M MgSO_4_ (Carl Roth GmbH, Karlsruhe, Germany) and 1M CaCl_2_ (Merck, Darmstadt, Germany) were added to M9 salts (5×). M9 medium was also supplemented with a 1 µg/mL aqueous solution of Thiamine, and the final volume was adjusted to 1L with deionized water. The 20% glucose solution was filter-sterilized (0.45 µm, Carl Roth GmbH, Karlsruhe, Germany) and added separately to the sterilized medium.

#### Maintenance of Bacterial Strains

Bacterial glycerol stocks were prepared for both strains in 60% glycerol (Carl Roth GmbH, Kaiserslautern, Germany) by mixing bacterial cultures, pre-grown in LB medium, with glycerol (ratio 0.75:0.25) and storing them at −80 °C. For each experiment, bacterial precultures were prepared by inoculating 5 mL LB medium from the glycerol stocks followed by overnight incubation under shaking conditions at 180 rpm at 37 °C. Prior to each MIC experiment, these precultures were diluted to an OD_600_ of 0.1 with LB broth and incubated again to reach an OD_600_ of 0.5 (BioPhotometer plus, Eppendorf, Hamburg, Germany) (main culture).

### 3.3. Antimicrobial Activity of Azithromycin and Menadione

Standard micro-broth dilution assays were performed to determine the antimicrobial activities of azithromycin (AZM) and menadione (MEN) against AZMr- *E. coli* DH5α and planktonic *P. aeruginosa* in 96-well plates [69]. Stock solutions of azithromycin (Apollo Scientific Ltd., Stockport, UK) and menadione (Sigma-Aldrich, Taufkirchen, Germany) were prepared in absolute ethanol and stored at 4 °C for up to 2 weeks. 5.5 × 10^5^ CFU mL^−1^ from the main culture were added to each well of the 96-well plate and treated with serially diluted AZM and MEN (in LB medium), ranging from 2 to 1024 µg mL^−1^.

As controls, 5.5 × 10^5^ CFU mL^−1^ in LB broth and LB medium blank were used. After 18 h static incubation at 37 °C, the inhibitory concentration was determined as the last clear well that had a comparable OD_600_ value to the sterile LB medium (OD_600_ ~ 0.04). After a 60 s shaking step, OD_600_ was measured using a Tecan Reader infinite M-Plex 200 (Tecan GmbH, Crailsheim, Germany).

#### 3.3.1. Checkerboard Assay-MIC

Interactions between azithromycin and menadione were studied by the checkerboard microdilution method (8 × 12 design) in 96-well flat-bottom microtiter plates in an LB medium. Menadione was added to columns A1–A10, starting from its minimum inhibitory concentration (64 µg/mL for AZMr-*E. coli* DH5α and 62.5 µg/mL planktonic *P. aeruginosa*) and then diluted vertically with LB broth. Azithromycin was added to rows A1–H1, starting from 256 µg/mL for AZMr-*E. coli* DH5α and 62.5 µg/mL for planktonic *P. aeruginosa* and then diluted horizontally with LB broth. Wells from column 12 were left for growth control, and column 11 was used as sterility control (LB medium blank). Then, 5.5 × 10^5^ CFU mL^−1^ cells from the main culture were added to each well of the 96-well plate. After 18 h incubation at 37 °C, the OD_600_ was measured using a Tecan Reader infinite M-Plex 200 (Tecan GmbH, Crailsheim, Germany). The lowest concentration of AZM combined with the lowest concentration of MEN showing no bacterial growth, was regarded as the MIC of the combination (AZM/MEN).

The fractional inhibitory concentration index (FICI) of the combinations was calculated based on Loewe’s additivity [70]:

(1) $$FICI=\frac{{MIC}_{AZM+MEN}}{{MIC}_{AZM}}+\frac{{MIC}_{MEN+AZM}}{{MIC}_{MEN}}$$

MIC_AZM+MEN_ is the MIC of azithromycin and menadione in combination, MIC_AZM_ is the MIC of azithromycin alone, and MIC_MEN_ is the MIC of menadione alone. Drug interactions were defined as synergistic if FICI ≤ 0.5, no interaction if 0.5 < FICI ≤ 4, and antagonistic if FICI > 4 [31].

#### 3.3.2. LC-MS Analysis

A Liquid Chromatography/Mass Spectrometry (LC/MS) (LC/ESI HR QTOF MS 6545 Spectrometer, Agilent Technologies, Santa Clara, CA, USA) method was developed using an HPH-C18 column with an acetonitrile/formic acid mobile phase for the identification and coarse concentration determination of MEN and AZM. The analysis was conducted in positive ionization mode, allowing for the detection of the exact masses of the positively charged ions [M + H]^+^ for both analytes (748.5085 Da for AZM and 172.0524 Da for MEN). The analyte concentrations were adjusted to antibacterial levels, with AZM and MEN prepared at 128 µg/mL and 16 µg/mL, respectively, in the mobile phase. Dilutions of stock solutions (10.24 mg/mL for both AZM and MEN) were used to prepare the mixtures. Each sample was analyzed in duplicate, and extracted ion chromatograms (ESI EIC) were used to calculate the average peak areas of the analytes. The data were processed using MassHunter software (version B 7.0 and version 10.1) to extract exact masses and compare area integrals of the analytes in the mixture.

### 3.4. MBEC Assay

A minimum biofilm eradication concentration (MBEC) assay was carried out to determine the antibiofilm potency of azithromycin and menadione. The assay was performed using the MBEC Biofilm Inoculator 96-well plates and protocol from Innovotech Inc. (Edmonton, AB, Canada) [35]. Suspensions of *P. aeruginosa* were diluted from the overnight culture to an OD_600_ of 0.1 with LB broth and incubated again to reach an OD_600_ of 0.5 (BioPhotometer plus, Eppendorf, Hamburg, Germany). Then, 2 × 10^5^ CFU mL^−1^ cells (150 µL inoculum/well) were added to the 96-well plate, the plate was covered with the MBEC lid and incubated at 37 °C for two and a half days, gently shaking with 3–5 rocks per minute. After 2.5 days, pegs with the biofilms grown at their surface were exposed to azithromycin and menadione. For this, the MBEC lid was added to a 96-well plate (challenge plate) that had the diluted solutions of AZM and MEN in LB medium, ranging from 2 to 1024 µg mL^−1^. The challenge plate was incubated at 37 °C for another 18 h, shaking with 3–5 rocks per minute. Afterwards, three parameters were measured:

MBEC determination based on turbidity: After treating the biofilms with different concentrations of azithromycin and menadione, the challenge plate was removed from the incubator, and the MBEC lid was transferred to a fresh 96-well plate containing 200 µL of fresh LB medium (recovery plate). The pegs were allowed to equilibrate in the recovery plate for 30 min and were subsequently sonicated for an additional 30 min to dislodge the biofilms into the wells of the recovery plate. To determine the MBEC, the recovery plate was closed with a sterile 96-well plate lid and incubated statically for another 18 h at 37 °C. The MBEC was defined as the last clear well with an OD_600_ value comparable to sterile LB medium OD_600_ ~ 0.04), which was measured using a Tecan Reader infinite M-Plex 200 (Tecan GmbH, Crailsheim, Germany).

log10 calculation based on viable counts: Following sonication, serial dilutions were prepared in sterile 0.9% NaCl solution from the biofilms dislodged from the pegs treated with 64–1024 µg/mL of azithromycin and menadione. These dilutions were spot-plated onto LB agar plates, which were then incubated statically at 37 °C for 18 h. The next day, colonies grown on the plates were counted to calculate the log10 reduction [log10 (CFU/peg)] in treated pegs compared to untreated controls. This allowed for an assessment of the effectiveness of azithromycin and menadione treatments in reducing biofilm viability.

MBC: The minimum bactericidal concentration (MBC) of azithromycin and menadione was determined in the challenge plate, as described in the protocol from Innovotech. First, a fresh sterile 96-well plate was filled with 180 µL LB medium (MBC plate). After the MBEC lid was removed from the challenge plate, 20 µL from each well of the challenge plate was added to the corresponding wells of the MBC plate. The plate was incubated statically for 18 h at 37 °C. The MBC was determined as the last clear well that had a comparable OD_600_ value to the sterile LB medium (OD_600_ ~ 0.04). OD_600_ was measured using a Tecan Reader infinite M-Plex 200 (Tecan GmbH, Crailsheim, Germany).

#### 3.4.1. Checkerboard Assay-MBEC

MBEC of combinations of azithromycin and menadione was determined the same as described above, except for the challenge plate. For the challenge plate, a checkerboard assay in LB medium was prepared. Azithromycin was added to columns A1–A10, starting from its MBEC concentration (256 µg/mL) and then diluted vertically with LB broth. Menadione was added to rows A1–H1, starting from its MBEC concentration (512 µg/mL) and then diluted horizontally with LB broth. Wells from columns 11 and 12 were left for growth control.

#### 3.4.2. Scanning Electron Microscopy (SEM)

SEM was performed to visualize the biofilms grown on untreated pegs and pegs treated with MBEC of menadione and azithromycin. After exposing the biofilms to the challenge plate, the pegs of interest were broken at the base of the peg from the MBEC lid with sterilized pliers. Pegs were placed in empty vials and the biofilms were fixed for 48 h by adding 5% (*v*/*v*) glutaraldehyde solution (Electron microscopy science, Hatfield, UK) into each vial (enough volume to cover the peg entirely). Afterwards, the fixative was discarded, and the pegs were left to dry for 24 h. Pegs were then placed vertically on aluminum stubs (the base of the peg) using double-sided carbon tape. Imaging was carried out using FEI (Hillsboro, OR, USA) Quanta 400 FEG in low vacuum mode (100 Pa) using a large field detector (LFD) and acceleration voltages of 5 kV and 10 kV at a working distance of 7.5–13.0 mm.

### 3.5. Reactive Oxygen Species in Bacteria

The generation of ROS was evaluated by 2′,7′ dichlorodihydrofluorescein diacetate (DCFH-DA) (Sigma-Aldrich, Taufkirchen, Germany). DCFH-DA is a cell-permeable, non-fluorescent probe, which is cleaved by intracellular esterases at the two ester bonds, producing 2′,7′ dichlorodihydrofluorescein (H_2_DCF). H_2_DCF can be converted via oxidation by ROS into the fluorescent product, 2′,7′ dichlorofluorescein DCF [71,72]. This assay indicates the general oxidative activity incorporating all ROS species such as for example H_2_O_2_, NO·, lipid peroxides, singlet O_2_ or O_2_· [73,74]. The protocol was adapted from [75].

A bacterial suspension with an OD_600_ of 1.0 was prepared in LB broth from an overnight pre-culture. Next, 2 mL of this suspension was added to each reaction tube, and cells were washed twice via centrifugation for 5 min at 10,000× *g* and resuspended in sterile DPPS (Gibco Invitrogen, Grand Island, NE, USA). Afterwards, bacteria were incubated with DCFH-DA at a final concentration of 10 µM, at 37 °C, 180 rpm for 30 min in the dark. After 30 min, the bacteria were washed using centrifugation for 5 min at 10,000× *g* and resuspended two times in sterile DPPS. Then, bacteria were treated with menadione and azithromycin for 4 and 12 h while incubating at 37 °C, 180 rpm. Finally, the fluorescence of the oxidized product DCF was measured using the Tecan Reader infinite M-Plex 200, at λ_ex_ = 488 nm and λ_em_ = 535 nm.

### 3.6. mCherry Leakage from E. coli Cells

AZMr-*E. coli* DH5α was inoculated with minimal medium and grown at 37 °C, 180 rpm, for about 2 days. Next, this pre-culture was diluted to an OD_600_ of 0.1 to adjust the number of cells used for inoculation of the test cultures. Then, 4 mL of this suspension was added into each reaction vial and cells were treated with different concentrations of menadione, ranging from 8 to 64 µg mL^−1^, for 2, 4, and 24 h. Every 2, 4, and 24 h, 1 mL bacterial suspension was taken from each test vial, centrifuged for 20 min at 10,000× *g* and the supernatants were collected. The fluorescence of mCherry leaked into the supernatant was measured by using the Tecan Reader infinite M-Plex 200 at an excitation wavelength of 587 nm and emission wavelength of 625 nm. As a positive control, cells grown over 2, 4, and 24 h were lysed for 30 min with a 9% Triton-X 100 solution (20 µL per 1 mL cell suspension). As a negative control, the same protocol was applied to the same AZMr-*E. coli* DH5α strain, which does not express the mCherry protein. To calculate the concentration of mCherry leaked in the supernatant over time, we prepared a calibration curve of pure mCherry in a minimal medium, starting from a concentration of 9.12 µM (Supplementary Figure S4). The Histidine-tagged mCherry released into the supernatants was also visualized by incubating the supernatants overnight with His-Ni agarose beads (ROTI^®^Garose-His/Ni Beads, Carl Roth GmbH, Karlsruhe, Germany) in a ratio of 1:5. These His-Ni affinity agarose beads were then visualized using a fluorescence microscope (Keyence BZ-X810, Keyence Deutschland GmbH, Leipzig, Germany).

### 3.7. Cell Culture

In this study, two human airway cell lines, Calu-3 and A549 cells, representing the bronchial and the alveolar epithelium, respectively, were used to probe the cytotoxicity of menadione and azithromycin.

*Calu-3* (HTB-55^TM^) epithelial cells [76] were obtained from the American Type Culture Collection (ATCC; Rockville, MD, USA) and maintained in a humidified incubator (37 °C, 5% CO_2_, and pH 7.4). Cells were nurtured with minimal essential medium (MEM) (Gibco Invitrogen, Grand Island, NE, USA), supplemented with 10% fetal bovine serum (FBS) (PAN-Biotech GmbH, Aidenbach, Germany), 1% sodium pyruvate (Life Technologies, Grand Island, NE, USA), 1% non-essential amino acids (Life Technologies, Grand Island, NE, USA), and 50 U/mL Penicillin and 50 µg/mL Streptomycin (Gibco Invitrogen, Grand Island, NE, USA). Sub-cultivation of the cells and cell maintenance at the air-interface conditions (AIC) was performed as previously described [54]. In brief, cells were seeded at a density of 10^5^ cells/mL in transwell plates with membrane inserts (Greiner Bio-One GmbH, Frickenhausen, Germany). The plates were filled with 1500 µL medium in the basolateral compartment and 500 µL medium in the apical compartment. Cells were allowed to adhere to the apical transwell membrane covered with medium for 24 h. Then, the apical medium was removed, and the cells were cultivated at AIC conditions for up to 21 days to promote cell differentiation.

*A549* (ACC107) cells [77] were obtained from the German Collection of Microorganisms and Cell Cultures (DSMZ, Braunschweig, Germany) and maintained in a humidified incubator (37 °C, 9% CO_2_, and pH 7.4). Dulbecco’s minimal essential medium (DMEM) (Gibco Invitrogen, Grand Island, NE, USA), supplemented with 10% fetal bovine serum (PAN-Biotech GmbH, Aidenbach, Germany), was used to grow and perpetuate the cells. A549 cells were sub-cultivated when they reached a confluence of around 70% by trypsinizing them for 5 min with a 0.05% trypsin solution containing 0.02% EDTA (PAN Biotech, Aidenbach, Germany).

### 3.8. Membrane Integrity Assay

The membrane integrity of Calu-3 and A549 cells was determined based on the activity of lactate dehydrogenase (LDH) in the cell culture medium. To perform the assay, both cell lines were seeded at a density of 10^5^ cells/mL (cell counter Innovatis Casy, Reutlingen, Germany) in black 96-well plates and allowed to adhere for 24 h prior to further incubation with test compounds.

Then, 100 µL of menadione and azithromycin solutions, respectively, was added to each well starting from a concentration of 1024 µg/mL and diluted with cell culture medium to 2 µg/mL, horizontally in the plate. Cells were exposed for 24 h to the test compounds and incubated at the respective aforementioned incubation conditions. The CytoTox-ONE Homogeneous Membrane Integrity Assay (Promega GmbH, Walldorf, Germany) was used according to the manufacturer’s instructions. After 24 h, 100 µL of the assay reagent was added to each well and incubated for 10 min at RT in the dark. After adding 50 µL of stop solution, fluorescence was recorded with a Tecan microplate reader (TECAN Spark multimode plate reader, Tecan Austria GmbH, Grödig, Austria) at an excitation wavelength of 560 nm and an emission wavelength of 590 nm. Background fluorescence of the cell-free medium was subtracted from the recorded values. Membrane integrity was related to a positive control (cells treated with 9% Triton X-100).

### 3.9. Reactive Oxygen Species in Calu-3 Cells

The ability of menadione, at the determined relevant antibacterial concentrations, to induce reactive oxygen species (ROS) in Calu-3 cells was evaluated by using 2’,7’-dichlorodihydrofluorescein diacetate (DCFH-DA) staining. The protocol was adjusted from [78]. In brief, Calu-3 cells, grown at an air interface and differentiated for 21 days were exposed to the test compounds by adding them at different concentrations in the basolateral medium of the transwell. Next, cells were kept in the incubator for 24 h, at 37 °C, 5% CO_2_. Upon 24-h exposure, Calu-3 cells were collected from the apical compartment using 3x washing with 500 µL DPBS (Sigma-Aldrich, Steinheim, Germany), transferred in tubes (Eppendorf, Greiner Bio-one GmbH, Frickenhausen, Germany) and centrifuged for 3 min, 200 rcf, 20 °C. Afterwards, 500 µL of fresh DPBS was added to the tubes and cells were incubated for 30 min in 5% CO_2_ at 37 °C with 10 µM DCFH-DA. After 30 min, cells were gently centrifuged again (200 rcf) for 3 min at 20 °C. The supernatant was aspirated to remove excess dye, and the cells were resuspended in fresh DPBS, transferred into black 96-well plates (200 μL/well), and the fluorescence of the samples was determined (excitation: 488 nm, emission: 530 nm) using a spectrophotometer (TECAN Spark multimode plate reader, Tecan Austria GmbH, Grödig, Austria).

## 4. Conclusions and Outlook

This study focuses on the ability of menadione to potentiate the activity of azithromycin against planktonic cells and biofilms of *P. aeruginosa,* taking into consideration its mechanism of action and the impact of its antibacterial concentrations in pulmonary cell models. We demonstrated that adding only 0.5 µg/mL menadione to azithromycin led to a four-fold reduction in the inhibitory concentration of azithromycin against planktonic *P. aeruginosa*. While menadione alone showed limited antibiofilm activity, it could reduce the antibiofilm concentration of azithromycin by two-fold (MBEC 128/16 µg/mL), acting this way as an adjuvant in eradicating the biofilms of *P. aeruginosa.* Menadione induced the generation of ROS species in both AZMr-*E. coli* DH5α and *P. aeruginosa* at its respective MIC and subMIC concentrations. By measuring the release of mCherry from the membrane of the model AZMr-*E. coli* DH5α strain into the supernatant, we confirmed that menadione disturbs the bacterial cell membrane. Thus, we could conclude that the antibacterial mechanism of menadione involves both ROS formation as well as membrane disruption. Future research utilizing fluorescently labelled *P. aeruginosa* would be an excellent approach to validate and expand on our findings on the effect of menadione on bacterial membrane integrity. Further, we tested whether these antibacterial concentrations of menadione are harmful to lung epithelial cells, by assessing their impact on the membrane integrity of Calu-3 and A549 cells. We found that MEN induced cytotoxicity in both cell lines at concentrations > 256 µg/mL, albeit not at the determined antibacterial levels (≤64 µg/mL). In addition, MIC concentrations of menadione caused significantly elevated ROS generation in Calu-3 cells. Nevertheless, at the inhibitory combination with azithromycin (15/0.5 µg/mL) against *P. aeruginosa*, ROS generation in Calu-3 cells was not significant. All in all, our results suggest that menadione is a promising compound for increasing the potency of azithromycin against *P. aeruginosa* infections and the potential facilitation of its internalization into the bacteria. Nonetheless, an accurate assessment of its cellular effects at relevant antibacterial concentrations is essential for ensuring the safe use of menadione in complementing antibiotic therapy. Further studies in more complex and clinically relevant settings can be performed to evaluate the efficacy and safety of menadione as an antibiotic adjuvant. For instance, the use of CFBE41o–cell lines derived from cystic fibrosis bronchial epithelial cells would provide insights into the interaction between menadione, azithromycin and the host cell environment, particularly in the context of cystic fibrosis [79]. In addition, the simultaneous assessment of menadione’s efficacy in eradicating biofilms and its safety on host cells would enable researchers to study the dual effects of menadione on bacteria and mammalian cells in a more realistic, physiologically relevant setting. For this, there are several approaches that can be used, like 3D-printed biofilm models layered on mammalian cells [80] or 3D epithelial co-culture models, where *P. aeruginosa* is grown alongside human epithelial cells [81]. Another approach would be the development of hydrogels embedding bacteria, which provide a controlled microenvironment to simulate the chronic infection niche and allow for the real-time assessment of the antibacterial activity and the cytotoxic effect on host cells [82].

## Figures and Tables

**Table 1:** MIC (µg/mL) of azithromycin and menadione, alone and combined, against AZMr-*E. coli* DH5α and *P. aeruginosa.* Both compounds inhibit bacterial growth individually; however, when combined (azithromycin/menadione) MIC is significantly reduced. Against *P. aeruginosa*, adding only 0.5 µg/mL menadione, a four-fold reduction in the inhibitory concentration of azithromycin was achieved. The FICI of AZM/MEN combinations is ≤ 0.5 in both strains, which indicates a synergistic interaction between the compounds. The experiment was performed in triplicate (N = 3).

Bacterial Strain	Azithromycin	Menadione	Azithromycin/Menadione	
	MIC (µg/mL)	MIC (µg/mL)	MIC (µg/mL)	FICI
AZMr-*E. coli* DH5α	512	64	128/16	0.5
*P. aeruginosa*	62.5	62.5	15/0.5	0.248

**Table 2:** The MBEC (µg/mL) of azithromycin and menadione, alone and combined, against mature biofilms of *P. aeruginosa*. MBEC of azithromycin is 256 µg/mL. When combined with menadione (azithromycin/menadione) azithromycin exhibits a lower MBEC as compared with azithromycin alone. In the presence of 16 µg/mL menadione, a two-fold reduction in the antibiofilm concentration of azithromycin is achieved. Experiment was performed in triplicate (N = 3).

Bacterial Strain	MBEC (µg/mL)		
	Azithromycin	Menadione	Azithromycin/Menadione
*P. aeruginosa*	256	512	128/16

**Table 3:** Characteristics of the strains used in this study.

Strain	Resistance	Plasmid	Fluorescence	Source	Abbreviation in Text
drug-resistant *E. coli* DH5α	ermB	pLp3050sNuc	mCherry	NEB, Art.No.C2987	AZMr-*E. coli* DH5α
*Pseudomonas aeruginosa* PAO1	-	-	-	DSM 22644	*P. aeruginosa*

## Data Availability

The data are not publicly available due to privacy restrictions.

## Supplementary Materials

The following supporting information can be downloaded at: https://www.mdpi.com/article/10.3390/antibiotics14020163/s1, Figure S1: Chemical structure and extracted ion chromatograms of azithromycin and menadione. Figure S2: Minimum bactericidal concentration in biofilms treated with azithromycin and menadione. Figure S3: His/Ni agarose beads treated with supernatants containing the released mCherry. Figure S4: Calibration curve of mCherry in minimal medium.
