# Supplementary material for: Menadione as Antibiotic Adjuvant Against P. aeruginosa: Mechanism of Action, Efficacy and Safety
Source: Antibiotics (Basel). 2025 Feb 7;14(2):163. doi: 10.3390/antibiotics14020163 (PMC11851977; doi:10.3390/antibiotics14020163)
Supplement: Supplementary file 1 [file antibiotics-14-00163-s001.zip › antibiotics-3447939-supplementary.pdf]

Menadione as Antibiotic Adjuvant Against *P. aeruginosa*: Mechanism of Action, Efficacy and Safety

Kristela Shehu [1,2], Marc Schneider [1,*] and Annette Kraegeloh [2,*]

[1] Department of Pharmacy, Biopharmaceutics & Pharmaceutical Technology, Saarland University, 66123 Saarbrücken, Germany; kristela.shehu@leibniz-inm.de

[2] INM—Leibniz Institute for New Materials, 66123 Saarbrücken, Germany

* Correspondence: marc.schneider@uni-saarland.de (M.S.); annette.kraegeloh@leibniz-inm.de (A.K.)

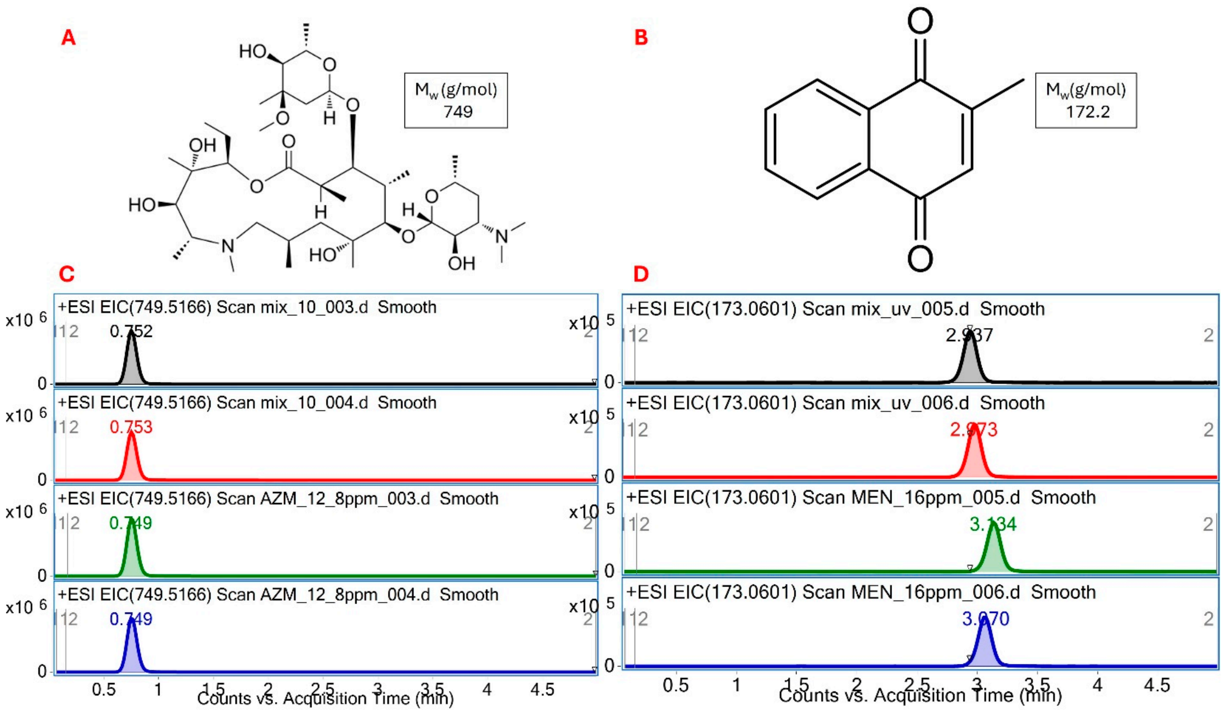

Supplementary Figure S1. (A) Chemical structure and molecular weight (749 g/mol) of azithromycin (AZM). **(B)** Chemical structure and molecular weight (172.2 g/mol) of menadione (MEN). **(C)** Extracted ion chromatograms (EIC) of azithromycin at 749.5166 m/z for the azithromycin-menadione mixture (mix_10_003 and mix_10_004) and azithromycin standards (AZM_12_8ppm_003 and AZM_12_8ppm_004). Retention times for azithromycin were consistent at approximately 0.749–0.753 minutes. **(D)** Extracted ion chromatograms (EIC) of menadione at 173.0601 m/z for the azithromycin-menadione mixture (mix_uv_005 and mix_uv_006) and menadione standards (MEN_16ppm_005 and MEN_16ppm_006). Retention times for menadione were consistent at approximately 2.937–3.004 minutes. Data was analyzed using LC/MS with an HPH-C18 column, and average peak areas were calculated via ESI EIC to confirm the absence of new compound formation in the mixture.

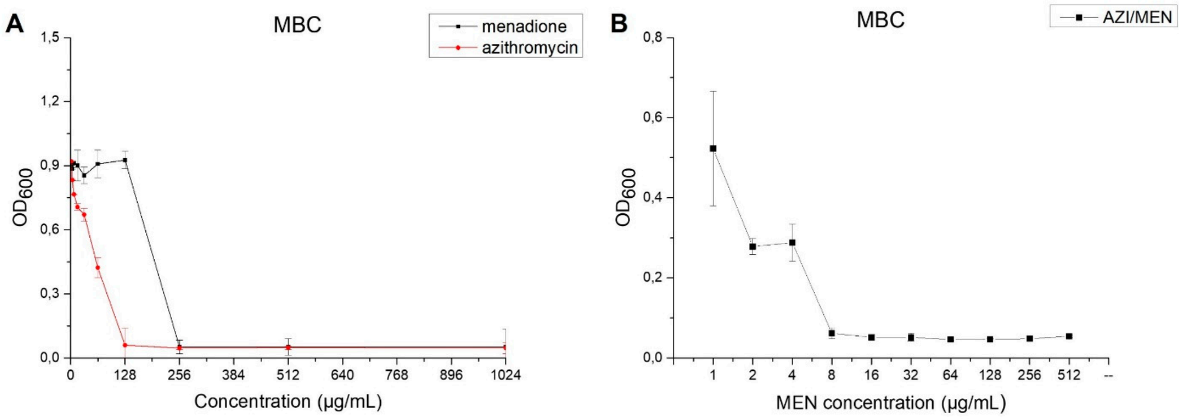

Supplementary Figure S2. Minimum Bactericidal Concentration (MBC) determined in biofilms treated with A) azithromycin or menadione at the given concentrations and B) 128 µg/mL azithromycin combined with menadione at the concentrations indicated on the x-axis. We determined an MBC of 128 µg/mL for azithromycin, 256 µg/mL for menadione and 128/8 µg/mL for combined (as in the main text, section 2.2.1).

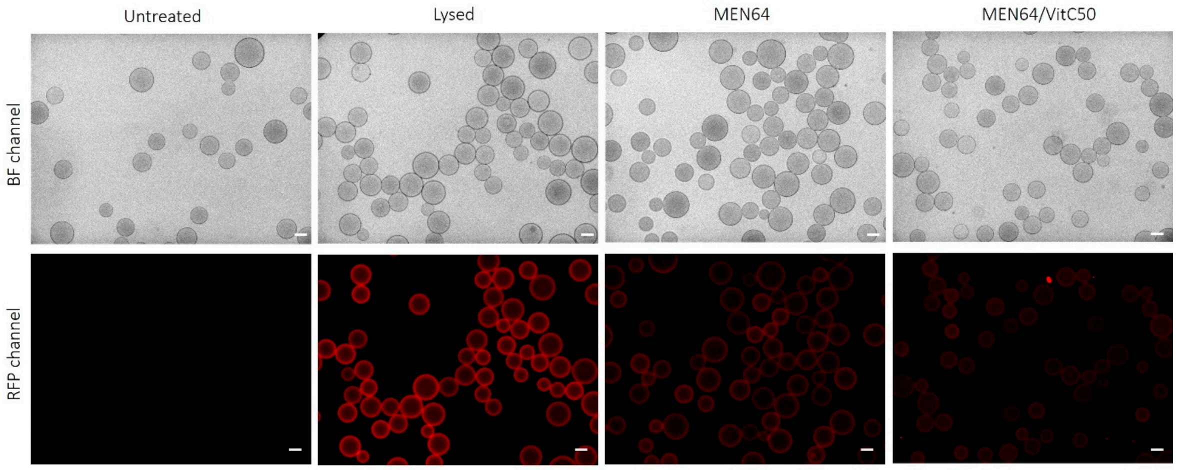

Supplementary Figure S3. His/Ni agarose beads treated with supernatants containing mCherry released from AZMr-*E. coli* DH5α under the indicated conditions. Top: bright field images, bottom: fluorescence images (RFP-channel). Scale bar 20 µm.

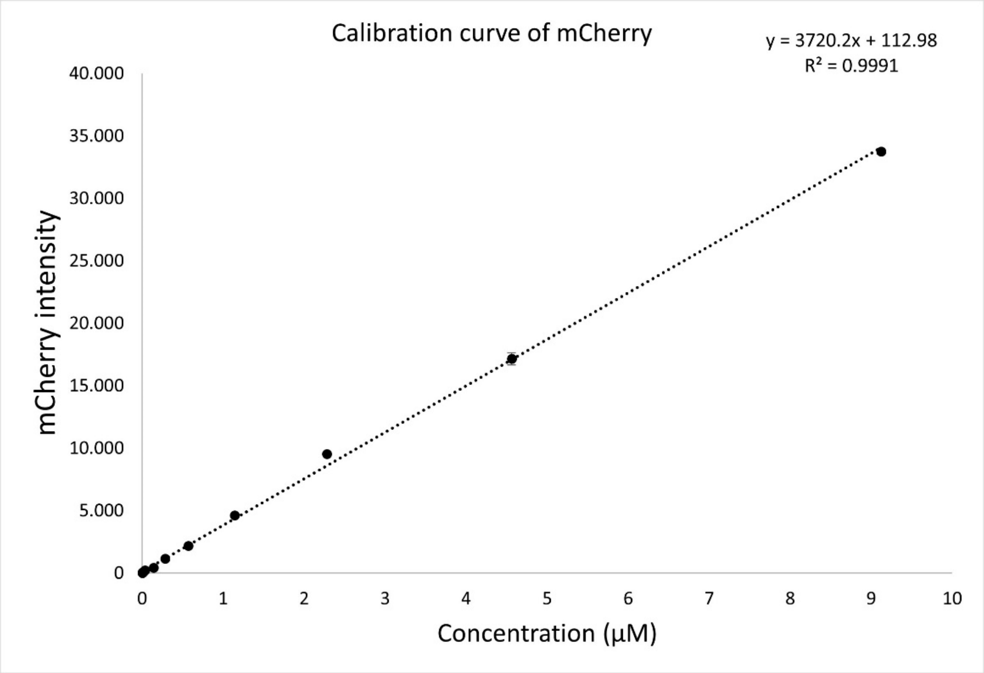

Supplementary Figure S4. Calibration curve of mCherry in minimal medium at concentrations of 0.0003-9.1µM (n=3). The equation was used to calculate mCherry concentrations based on fluorescence intensity.
